# Patient Perspectives on Healthcare Utilization During the COVID-19 Pandemic in People with Multiple Sclerosis—A Longitudinal Analysis

**DOI:** 10.3390/healthcare13060646

**Published:** 2025-03-16

**Authors:** Heidi Stölzer-Hutsch, Dirk Schriefer, Joachim Kugler, Tjalf Ziemssen

**Affiliations:** 1 Department of Neurology, Center of Clinical Neuroscience, Medical Faculty and University Hospital Carl Gustav Carus, TUD Dresden University of Technology, Fetscherstr. 74, 01307 Dresden, Germany; heidi.stoelzer-hutsch@ukdd.de (H.S.-H.); dirk.schriefer@ukdd.de (D.S.); 2 Institute and Policlinic for Occupational and Social Medicine, Medical Faculty and University Hospital Carl Gustav Carus, TUD Dresden University of Technology, Fetscherstr. 74, 01307 Dresden, Germany; joachim.kugler@tu-dresden.de

**Keywords:** multiple sclerosis, COVID-19 pandemic, healthcare accessibility, patient perception

## Abstract

Background/Objectives: The COVID-19 pandemic disrupted healthcare systems globally, altering the management of chronic conditions like multiple sclerosis (MS) and interrupting the regular monitoring and support that people with MS (pwMS) typically need. The aim of this study was to examine changes in the utilization of MS healthcare resources over various periods during the COVID-19 pandemic in 2020 and 2021, and to assess how these changes affected the perceptions of pwMSregarding their care. Methods: A longitudinal survey study was conducted at the MS Center at the University Hospital Dresden, Germany, involving four survey periods from April 2020 to December 2021. The study assessed the use of healthcare resources, including consultations with specialists, the use of rehabilitative therapy facilities, and unmet healthcare needs, across various phases of the pandemic, encompassing both lockdown and less restrictive periods. Results: At the onset of the pandemic in April 2020, during the first lockdown, 750 questionnaires were evaluated. While most pwMS reported consistent medical care compared with pre-pandemic levels, 19.2% had fewer general practitioner visits, and 10.6% fewer neurologist visits. During the follow-up survey periods, the use of medical care generally remained stable, although there were notable reductions reported by a subset of participants. Conclusions: The findings suggest that medical and therapeutic care for pwMS in Germany remained largely accessible during the COVID-19 pandemic in 2020 and 2021. However, the study also reveals certain gaps in care that may be addressed by incorporating digital technologies into medical care and rehabilitation, potentially enhancing the management of healthcare during future pandemics or similar situations.

## 1. Introduction

To prevent the spread of the coronavirus disease 2019 (COVID-19) during the pandemic in 2020 and 2021, restrictive measures such as social distancing, bans on public events, nighttime curfews, travel bans, self-isolation, and ultimately lockdowns—comprehensive measures restricting people’s mobility and closing various establishments—were implemented. While these interventions were necessary to control the pandemic, they also posed significant challenges for the continuous care required by individuals with chronic diseases like multiple sclerosis (MS) [1]. Continuous care is essential for people with MS (pwMS) to promptly address clinical changes, but the pandemic restrictions disrupted their access to regular medical appointments, therapies, and rehabilitation services.

MS is a chronic inflammatory, progressive disease of the central nervous system with a largely unclear etiology [2]. It typically affects several functional systems of the human body, which can lead to a wide range of different symptoms and a very individualized course of the disease. The main manifestations are cognitive, sensory, and motor deficits. In most cases, several symptoms occur simultaneously, which impairs quality of life and makes activities of daily living more difficult. The first manifestation of the disease usually occurs in young adulthood, usually between the ages of 20 and 40. This makes MS the most common inflammatory neurological disease in younger adults [2]. Worldwide, 2.8 million people suffer from MS—and the trend is rising [3,4].

The onset of the COVID-19 pandemic in March 2020 abruptly changed MS healthcare management [5]. PwMS cancelled their regular appointments [6,7] and reduced their physical activity [8]. Continuous medical care within patient-centered MS management is necessary for the comprehensive care of pwMS [9]. This includes regular outpatient appointments, disease-related relapse management, immunomodulatory treatment, rehabilitation programs, and MRI appointments [10]. Regular therapy and medical supervision are crucial for preventing disease progression, managing symptoms effectively, and optimizing long-term outcomes [11]. Consistent engagement with healthcare professionals ensures timely adjustments to treatment plans and supports the overall well-being of pwMS [11].

Due to COVID-19-related restrictions, challenges in accessing care for pwMS have been highlighted in numerous cross-sectional analyses [5,12,13,14,15,16]. Some longitudinal studies, mostly retrospective, were also able to show the impact of the pandemic on healthcare for pwMS over time [17]. The aim of this study was to depict changes in MS healthcare resource use over different time periods during the COVID-19 pandemic in 2020 and 2021 and in the perception of MS care by those affected. It therefore outlines the situation in April 2020, at the onset of the first lockdown, and the subsequent development of outpatient care from the patients’ perspective. This could provide valuable insights into the potential long-term vulnerability and adaptive capacity of the health system, providing information that could help to better assess the pandemic and its consequences, and ultimately enable more effective management of similar situations in the future.

## 2. Materials and Methods

### 2.1. Recruitment and Survey Periods

We conducted a longitudinal survey study at four different time periods at the outpatient Multiple Sclerosis Center at the Center of Clinical Neuroscience, Neurological University Clinic Carl Gustav Carus, Dresden, Germany. PwMS were invited to complete the initial survey during their routine examinations in April 2020 at the MS Center, which also marked the recruitment phase for the study. In all subsequent surveys, participants recruited during this period were asked to complete follow-up surveys.

**Figure 1:**
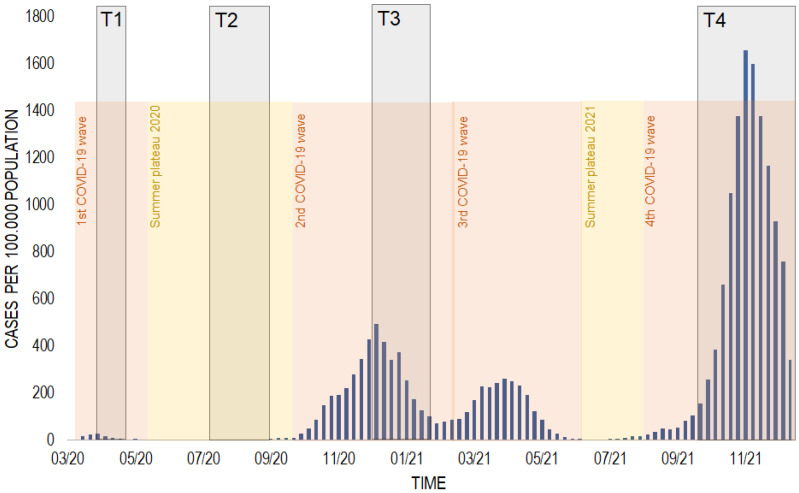
Time course of COVID-19 incidences in Saxony, Germany, over the survey periods in 2020 and 2021. COVID-19 incidences (blue), survey periods (grey), and phases of COVID-19 pandemic (orange and yellow). Survey periods: T1 = first survey from 6 April to 30 April 2020 (during first COVID-19 wave: first lockdown); T2 = second survey from 8 July to 31 August 2020 (during summer plateau 2020: low incidence); T3 = third survey from 1 December 2020 to 31 January 2021 (during second COVID-19 wave: partial lockdown and second lockdown; launch of COVID-19 vaccination campaign in Germany); T4 = fourth survey from 8 October to 31 December 2021 (during fourth COVID-19 wave: peak infections, expanded vaccination efforts, reintroduced public health measures). Data source: Robert Koch Institute.

The survey periods can be retrospectively labelled in individual phases of the COVID-19 pandemic, according to the classification of the Robert Koch Institute, Germany’s national public health agency [18]. Survey periods were as follows: T1 in April 2020 (first COVID-19 wave); T2 in July and August 2020 (summer plateau 2020); T3 from December 2020 to January 2021 (second COVID-19 wave); T4 in October to December 2021 (fourth COVID-19 wave) (see Figure 1). These phases reflect critical epidemiological and societal milestones during the pandemic in Germany. The study was approved by the Ethic Commission of the Technical University of Dresden.

### 2.2. The Survey Design

The surveys were designed to provide a comprehensive view of the challenges posed by the COVID-19 pandemic. They were based on items regarding medical and therapeutic MS care, as well as the needs of pwMS. Fifteen items were examined, grouped into the domains of healthcare resource use, patient perception, and unmet needs (see Appendix A Table A1). The items within the domain of healthcare resource use (No. 1–4) encompass changes in medical and therapeutic care during the COVID-19 pandemic, focusing on the frequency of visits to healthcare providers (general practitioner, neurologist) and the utilization of therapeutic treatments (physical therapy, occupational therapy, speech therapy, rehabilitation sports). Additionally, they assess levels of satisfaction with therapeutic care. The items within the domain of patient perception and unmet need (No. 5–15) explore the experiences of and challenges faced by patients during the COVID-19 pandemic. They assess the extent to which patients feel they are missing essential MS therapies, encounter unmet needs for support, or experience dissatisfaction with their medical care. The items also address preferences for medical advice, psychological support, telemedicine services, and participation in internet-based sports, as well as decisions regarding the continuation or modification of MS therapies due to the pandemic.

A total of nine items were administered in multiple survey periods, T1–T4, enabling a longitudinal analysis. Six items were only included in the fourth survey (T4), as this allowed for reflection on the past pandemic periods. A 2- to 4-point scale was chosen for response options (e.g., a 4-point scale with: do not agree at all, tend to disagree, tend to agree, fully agree). A standardized survey instrument was used as the foundation for the survey design [19], which was further supplemented with specific questions pertaining to MS (e.g., “How often have you been dissatisfied with the medical care you have received for your MS in recent days?”).

### 2.3. Statistical Methods

The distribution of response options for each survey item and period was described and visualized using 100% stacked bar charts, presenting both absolute and relative frequencies. These visualizations included data from both the full study cohort and the complete-case group, thereby providing a comprehensive overview of cross-sectional distributions and longitudinal changes.

To assess changes in survey items over time, longitudinal analyses were conducted using the complete-case group. The dependent variables included items No. 1, 5–9, 11, 13, and 15, representing all items that were assessed at multiple points in time, either T1–T4 or T1–T3 (see Appendix A Table A1). For ordinal variables, the Friedman test was applied, while binary variables were analyzed using Cochran’s Q test. Post-hoc comparisons were conducted with Bonferroni correction to identify significant differences between survey periods. These analyses were designed to evaluate temporal trends and provide insights into behavioral changes during different phases of the pandemic.

The associations between key survey items from the initial survey period (T1) and socio-demographic and disease-related factors were examined using logistic regression. The dependent variables included neurologist visits (item No. 1b), physiotherapy visits (item No. 1c), telemedicine preference (item No. 13), and online sports participation (item No. 15). Predictor variables in this analysis were age (categorical), Expanded Disability Status Scale (EDSS) score (categorical), gender, place of residence (urban district versus rural district), and immunotherapy (yes/no). Odds ratios with 95% confidence intervals were reported for all logistic regression analyses to assess the strength and precision of associations between the predictor variables and the dependent survey items.

Participants were classified based on their postal codes into urban districts (Leipzig, Chemnitz and Dresden in Saxony, Germany) and rural districts (ten districts in Saxony, Germany) to examine the impact of population density on patient behavior. Dichotomization was applied to some dependent variables to facilitate the logistic regression analysis. Specifically, for item No. 1 (visits to neurologists/physiotherapists), the categories “same” and “more frequent” were collapsed into a single category to focus on individuals with less frequent visits. Items 13 and 15 were maintained in their original binary format (yes/no).

The statistical significance threshold for all analyses was set at *p* < 0.05. All calculations were performed using IBM SPSS Statistics for Windows, version 29 (IBM Corp., Armonk, NY, USA).

## 3. Results

### 3.1. Participants

Surveys were distributed to 946 patients, with a response rate of 79.3% (n = 750) at T1 and approximately 45% for the follow-up surveys (see Table 1). It is important to note that the general characteristics of participants, such as age and disability level, tended to remain stable across all follow-up surveys (T2–T4), with only minimal variation. The complete-case group (n = 240) had a slightly older mean age compared to the groups of participants who did not take part in all of the surveys.

At T1, 16.1% (n = 152) of participants who had cancelled on-site appointments at the MS Center Dresden were registered. On average, these individuals were 50.5 years old (±13.24), with females comprising 73.0% of the sample. The median EDSS Score was 3 (interquartile range 2–6), and 56.8% of participants received immunomodulatory therapy.

### 3.2. Healthcare Resource Use

**Figure 2:**
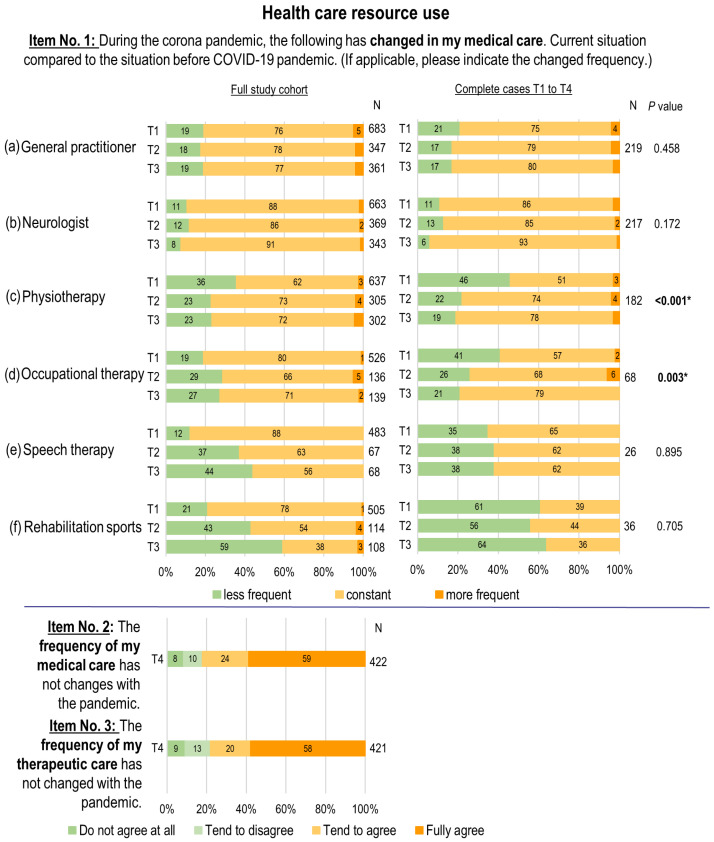
Key results: impact of the COVID-19 pandemic on healthcare resource use in MS care. T1 = first survey, April 2020 (first lockdown); T2 = second survey, July to August 2020 (summer plateau 2020); T3 = third survey, December 2020 to January 2021 (second lockdown); T4 = fourth survey, October to December 2021 (fourth COVID-19 wave); bold and asterisk denote statistical significance between two or more survey points.

The majority of participants did not experience any changes in the consultations of healthcare resources at T1 compared to the situation before the COVID-19 pandemic (range: 62% visits of physiotherapy to 88% visits of neurologists) (see Figure 2). Accordingly, most pwMS visited medical facilities in the first lockdown (T1) just as often as before the onset of the COVID-19 pandemic. With regard to visits to specialists, 19.2% of participants indicated a reduction in the frequency of consultations with general practitioners, while 10.6% reported a similar reduction in the frequency of consultations with neurologists. A reduction was noted in physiotherapy visits, with 35.8% (n = 228) of treatments used less frequently, followed by a reduction in occupational therapy (19.0%, n = 100) and speech therapy visits (12.2%, n = 59) compared to the situation before the onset of the COVID-19 pandemic.

The follow-up analysis from T1 to T3 revealed a significant increase in physiotherapy visits (*p* < 0.001) and occupational therapy visits (*p* = 0.003). For occupational therapy visits, this result relates to the complete-case group, which comprises a small number of participants. The post-hoc tests for the item “physiotherapy visits” showed an increase from T1 to T2 (adjusted *p* = 0.003) and from T1 to T3 (adjusted *p* = 0.001). The use of rehabilitation sports facilities showed a descriptive decline in visits from T1 to T3 in the full study cohort.

At T4, 17.7% (n = 75) of the participants stated that they had noticed a change during the COVID-19 pandemic (item No. 2). With regard to rehabilitative care (physiotherapy, occupational therapy, speech therapy, rehabilitation sports), 21.9% (n = 92) perceived a change during the COVID-19 pandemic (item No. 3). A total of 96.0% (n = 390) of participants at T4 reported being at least somewhat satisfied with their therapeutic care, while only 4.0% (n = 25) indicated being dissatisfied (item No. 4).

### 3.3. Patient Perceptions and Unmet Needs

**Figure 3:**
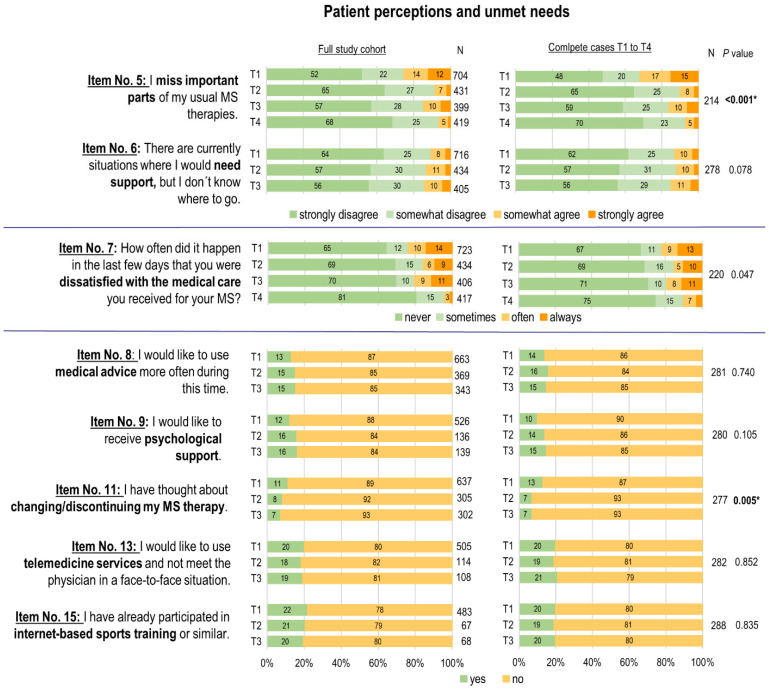
Key results: patient perceptions and unmet needs in MS care during the COVID-19 pandemic. T1 = first survey, April 2020 (first lockdown); T2 = second survey, July to August 2020 (summer plateau 2020); T3 = third survey, December 2020 to January 2021 (second lockdown); T4 = fourth survey, October to December 2021 (fourth COVID-19 wave); bold and asterisk denote statistical significance between two or more survey points.

At T1, 10.6% (n = 76) of respondents expressed a desire for more support (see Figure 3). In contrast, 76.3% (n = 552) were mostly satisfied with their MS care, while 23.7% (n = 171) were dissatisfied. A minority of 11.3% (n = 80) were considering changing their current MS treatment. One out of ten pwMS reported missing medical advice or psychological support. About one in five pwMS used internet-based sports training during the first lockdown (T1), and just as many preferred telemedicine services over in-person doctor visits.

In the follow-up analysis (T1–T4), pwMS were more likely to miss components of their therapy at T1 and T3 than at T2 and T4 (*p* < 0.001). These perceived limitations were most pronounced at T1 and T3, with 25.3% (n = 178) and 14.5% (n = 58) of respondents affected, respectively. Significant improvements were observed from T1 to T2 (adjusted *p* < 0.001), T3 (adjusted *p* = 0.015), and T4 (adjusted *p* < 0.001), as well as from T3 to T4 (adjusted *p* = 0.47).

PwMS were predominantly not dependent on additional support, although the proportion of those who missed support increased from T1 to T3, this change was not statistically significant (*p* = 0.078). PwMS were particularly dissatisfied with MS care at T1 (23.7%, n = 117) and T3 (20.5%, n = 83). However, it should be emphasized that in all analyzed periods, over 75% of participants were almost satisfied with their MS care situation and the proportion of satisfied respondents increased steadily over the course of the COVID-19 pandemic. By T4, reports of no dissatisfaction with MS care reached 80.8% (n = 337). From T1 to T3, the majority of participants did not miss medical advice (86.9% (n = 623) at T1 and 84.6% (n = 341) at T3) and most participants did not require additional psychological support (87.8% (n = 624) at T1 and 83.7% (n = 333) at T3) (see Figure 3). At T4, 9.7% (n = 41) reported receiving psychological support during the COVID-19 pandemic (item No. 10).

The possibility of discontinuing or changing their current MS treatment due to the pandemic situation was considered by a small proportion of participants. This proportion steadily decreased from 11.3% (n = 80) at T1 to 7.3% (n = 29) at T3. Ultimately, just 2.6% (n = 11) actually decided to discontinue or change their treatment (item No. 12). The majority of respondents did not participate in internet-based sports training from T1 to T3, despite the limited opportunities for rehabilitation and fitness sports during the COVID-19 pandemic. Most preferred attending face-to-face medical appointments over using telemedicine services. At T4, 7.3% (n = 31) of participants had actually participated in a telemedicine therapy session to avoid in-person consultations (item No. 14).

### 3.4. Evaluation of Influencing Factors in Relation to Four Key Items at T1 in April 2020

Participants aged 65 years and older were significantly less likely to visit their neurologist after the outbreak of the COVID-19 pandemic compared to younger individuals (OR 3.37; 95% CI 1.31–8.65; *p* = 0.012) (see Table 2; item No. 1b). The logistic regression analysis showed that pwMS taking MS medication were not less likely to see a neurologist compared with treatment-naive pwMS (OR 0.48; 95% CI 0.28–0.82; *p* = 0.008).

During the first lockdown (T1), women were significantly less likely to visit physiotherapy compared to men after the outbreak of the COVID-19 pandemic (OR 1.56; 95% CI 1.06–2.3; *p* = 0.023) (see Table 2; item No. 1c). Disability level (EDSS) and place of residence were not associated with changes in visits to neurologists or physiotherapists.

At the time of the first lockdown (T1), significantly fewer middle-aged and older patients participated in internet-based sports training compared to pwMS aged between 18 and 35 years (OR 3.28; 95% CI 2.23–4.83; *p* < 0.001 for middle-aged participants and OR 7.21; 95% CI 2.74–18.96; *p* < 0.001 for older participants) (see Table 2; item No. 13). PwMS with higher EDSS levels were significantly less likely to participate in internet-based sports activities compared to those with lower EDSS levels. In addition, pwMS living in rural districts participated less in internet-based sports training than pwMS living in urban districts (OR 1.76; 95% CI 1.23–2.50; *p* = 0.002).

PwMS living in rural districts were less likely to want to use telemedicine services than pwMS living in urban districts (OR 0.53; 95% CI 0.36–0.76; *p* < 0.001) (see Table 2; item No. 15). The factors of age, gender, disability, and MS medication had no influence on the decision to prioritize telemedicine services.

## 4. Discussion

The main objective of this study was to analyze changes in MS healthcare resource utilization during the COVID-19 pandemic in 2020 and 2021, as well as to understand patient perceptions of MS care in Germany. For this purpose, a longitudinal study was conducted at the outpatient MS Center of the Center of Clinical Neuroscience, Neurological University Clinic Carl Gustav Carus Dresden, spanning the critical periods of the pandemic, with surveys administered at four distinct intervals from the onset the COVID-19 pandemic onward: T1 during the first lockdown in April 2020; T2 in the summer of 2020, characterized by low COVID-19 incidence rates; T3 in the second lockdown of late 2020; and T4 at the end of 2021, by which time COVID-19 vaccinations had been available for nearly a year, though restrictions were still in place. Each survey reflects a different phase of the COVID-19 pandemic, capturing changes in public health measures and infection rates.

At the onset of the COVID-19 pandemic, pwMS experienced disruptions in both medical and therapeutic care. There was a perceived decrease in visits to general practitioners and neurologists, with 19.2% and 10.6% of pwMS, respectively, reporting fewer consultations than compared to before the pandemic. Despite these disruptions, the utilization of medical services remained stable or even improved during subsequent phases of the pandemic. The most notable reduction was observed in therapeutic services, particularly in physiotherapy during the first lockdown. Approximately a quarter of pwMS visited treatment facilities less frequently compared to before the pandemic. However, the need for additional care was rarely identified, and dissatisfaction with MS care remained low.

Information on the healthcare situation in Germany is available in the report from the Central Research Institute of Ambulatory Health Care (Zentralinstitut für die kassenärztliche Versorgung), which includes billing data and early insights from sixteen statutory health insurance physician associations for 2019 and the pandemic years 2020–2022. This report indicates a 23.4% decrease in visits to general practitioners and a 31.7% decrease in visits to neurologists in April 2020 within the general population [20]. The decreases observed in this study are lower than those reported nationally. International studies have reported a more significant decline in the use of medical services, ranging from 22% to 91% due to the onset of the COVID-19 pandemic [5,15,17]. This indicates that medical care for pwMS in Germany (Saxony) remained comparatively stable during the beginning of the COVID-19 pandemic. The ongoing prescription of MS-specific medications and antidepressants during the first and second lockdowns, compared to pre-pandemic levels, further indicates stability in MS care [10]. Additionally, patterns of care and treatment adjustments observed in the MS cohort were consistent with findings from other large-scale studies of pwMS during the pandemic, reinforcing the notion that the healthcare system adapted effectively to patient needs under challenging conditions [21]. Despite the ongoing contact restrictions from April to December 2020, the demand for medical consultations—whether digital or in-person—remained steady throughout the observation period (T1 to T3), suggesting that the MS Center was able to meet patient needs. A similar conclusion was drawn in a study involving 16 Italian MS centers [14]. At T4, only 7.3% of respondents reported using telemedicine services during 2020 and 2021, suggesting that fewer pwMS took advantage of this option. To enhance healthcare provision, it is crucial to explore the integration of telemedicine and digital biomarkers, which could better meet the evolving needs of patients [11,22]. Further investigation is needed to determine the most effective ways to implement telemedicine for patients and medical staff [23,24].

Most pwMS continued visiting therapeutic facilities in T1 at similar rates to pre-pandemic levels. However, 12.2% to 35.8% reported less frequent visits to occupational therapy, physiotherapy, or speech therapy during the first lockdown. Notably, physiotherapy usage decreased significantly, a trend supported by other studies [8,25,26,27]. The reduction in physiotherapy sessions during the first lockdown may have stemmed from therapeutic staff’s concerns about potentially endangering patients, their own health, and that of their families in an evolving situation [28,29]. However, from T1 to T3, T2 (*p* = 0.003) and T3 (*p* = 0.001) especially showed a significant increase in physiotherapy units compared to T1, which was a positive sign for physiotherapy care during the COVID-19 pandemic. Female pwMS reported using physiotherapy less frequently than male participants, with a significant difference observed during the first lockdown (T1). This may be partly explained by higher levels of COVID-19-related fear reported among women at the pandemic’s onset, as noted by Vogel et al. [15]. The present longitudinal analysis revealed heterogeneous utilization of therapies, with physiotherapy use increasing from T1 to T3, while occupational and speech therapy use declined; however, a smaller participant pool for the latter therapies may limit the data’s validity. On average, the utilization of various therapies was reduced for around a quarter of participants. As legal restrictions tightened, the reported utilization of rehabilitation sports decreased from 78.2% at T1 to 28% at T3, although this trend was not statistically significant in the complete-case group, which was only 39% at T1. This decline may have contributed to increased sedentary behavior, a trend also observed in the general population, although healthy individuals remained more active than those with pwMS [26,30,31]. It can be concluded that during periods of curfew, physical activity should not be overlooked, as it has been demonstrated that the quality of life of those who are more active is higher [30]. Telerehabilitation in the area of physiotherapy, occupational and speech therapy, but also in the field of rehabilitation sports, can ensure continuous further care in such exceptional situations. A meta-analysis showed that motor symptoms in particular could be addressed in pwMS within a telerehabilitation setting [32]. At T1, 21.9% of participants engaged in internet-based sports training, with no significant increase reported at T2 or T3. Notably, these programs reached mainly younger pwMS (ages 18–34) with lower levels of disability (EDSS < 3.5) living in urban areas, leaving the most vulnerable groups underrepresented. Matysiak et al. confirmed a greater interest in online sports among younger pwMS with lower degrees of disability in a survey conducted at a Polish MS center [33].

Even during the COVID-19 pandemic, pwMS felt adequately cared for, with about one in ten expressing a need for additional medical advice or psychological support during the first lockdown, and no significant changes noted from T1 to T3. These findings are supported by the results of several studies showing that there was no to less of an increase in mental health problems in pwMS during the pandemic in comparison to the general population [34,35]. During phases of higher restrictions (T1 and T3), components of MS therapy were more commonly missed, leading to increased dissatisfaction with MS care. In contrast, during periods of lower restrictions (T2 and T4), these components were less frequently missed. Dissatisfaction with MS care was low, with 80.8% of participants reporting no issues by late 2021 (T4). A high level of satisfaction was also reported in the Italian multicenter study by Altieri et al. in April/May 2021 [14]. Overall satisfaction with multiple sclerosis centers was 92%, and 93% of the participants reported maintaining or even increasing their confidence in their professional MS care [14].

When interpreting the results of this study, several limitations must be considered. The reliance on self-administered questionnaires introduces potential bias. While the first survey had a relatively high response rate, subsequent surveys likely suffered from selection bias, as seen in the gradual increase in average participant age and a decline in male respondents, particularly when comparing the first survey (T1) to later ones (T2, T3, and T4). Notably, only a quarter of the original participants completed all four surveys, with this group having the highest average age. Given that COVID-19 disproportionately impacted older populations, it is expected that interest in the study may have been higher in this demographic. Additionally, the results cannot be extrapolated to pwMS who are not closely monitored at specialized MS centers. Nevertheless, a key strength of the study is its longitudinal design, allowing for a comprehensive analysis across different phases of the COVID-19 pandemic. Additionally, comparisons between the full study cohort and the complete-case group revealed only minor differences in a few items, suggesting that the findings remain largely consistent and robust.

## 5. Conclusions

The surveys aimed to gain insights into the COVID-19 pandemic, particularly regarding healthcare delivery from the patients’ perspective. During the first lockdown, compared to before the start of the COVID-19 pandemic, changes in medical care were perceived by pwMS. The follow-up analysis showed consistent care during different periods of the pandemic and a high level of satisfaction with MS care. Targeted education regarding telemedicine and online sports programs will be of great importance in the future. This could help to lower the inhibition threshold for older people with MS and those living in rural areas to utilize such services. The use of digital technologies in medical care and rehabilitation could help to close identified gaps in care and better manage future pandemics or similar situations [36].

## Figures and Tables

**Table 1:** Sociodemographic and clinical characteristics of the participant groups at the different survey periods.

	Participants 1st Survey (T1)	Participants 2nd Survey (T2)	Participants 3rd Survey (T3)	Participants 4th Survey (T4)	Complete Cases T1 to T4
n/n_all_	750/946	441/946	411/946	422/946	240/946
Response rate; %	79.3	46.6	43.4	44.6	25.3
Age; years, mean (SD)	47.00 (13.05)	49.01 (13.71)	49.58 (13.32)	49.53 (13.18)	51.43 (13.42)
Range	19–85	19–85	19–85	19–85	19–85
Female; %	74.4	77.3	76.4	76.5	79.6
Place of Residence; %					
urban District	44.7	44.7	43.8	43.4	41.3
rural District	55.3	55.3	56.2	56.6	58.8
EDSS; median (IQR)	2.5 (1.5–4.5)	3.0 (1.5–5.0)	3.0 (2.0–5.0)	3.0 (1.5–5.0)	3.0 (2.0–5.5)
Disease duration; years, median (IQR)	10 (5–16)	10 (5–16)	11 (5–17)	10 (5–16)	12 (5–17.75)
Current immunotherapy; %	76.1	72.1	72.0	72.2	69.6
MS subtypes; %					
RRMS	81.5	79.4	79.6	80.1	80.4
SPMS	8.8	10.4	10.5	10.0	11.3
PPMS	8.9	9.3	9.0	9.5	7.9
CIS	0.8	0.9	1.0	0.5	0.4

The complete-case group includes participants answering the items in all survey periods. Abbreviations: EDSS = Expanded Disability Status Scale, RRMS = Relapse Remitted Multiple Sclerosis, SPMS = Secondary Progressive MS; PPMS = Primary Progressive MS; CIS = Clinically Isolated Syndrome.

**Table 2:** Impact of age, disability, gender, residence, and immunotherapy on changes in neurology and physiotherapy consultations during the first lockdown, and on internet-based sports training and telemedicine preferences at T1—analyzed using logistic regression.

Variable	Visits Neurologist Item No. 1b		Visits Physiotherapist Item No. 1c		Online Sports Item No. 15		Telemedicine Preference Item No. 13	
	OR	95% CI	OR	95% CI	OR	95% CI	OR	95% CI
Age								
young (18–34)	1	-	1	-	1	-	1	
middle-aged (35–65)	1.67	0.82–3.38	1.47	0.98–2.20	3.28 *	2.23–4.83	1.13	0.72–1.75
old (>65)	3.37 *	1.31–8.65	1.15	0.58–2.28	7.21 *	2.74–18.96	0.87	0.40–1.90
Disability (EDSS-Score)								
low (0–3.5)	1	-	1	-	1	-	1	
middle (4–6)	1.05	0.53–2.10	1.23	0.80–1.88	1.95 *	1.18–3.32	0.76	0.45–1.29
high (6.5–9)	1.45	0.67–3.18	1.49	0.90–2.47	5.62 *	2.22–14.27	1.66	0.95–2.87
Gender								
male	1		1	-	1	-	1	-
female	0.85	0.49–1.48	1.56 *	1.06–2.30	0.65	0.42–1.01	0.80	0.53–1.20
Residence								
urban districts	1	-	1	-	1	-	1	-
rural districts	1.42	0.85–2.36	0.82	0.59–1.14	1.76 *	1.23–2.50	0.53 *	0.36–0.76
Immune therapy								
none	1		1	-	1	-	1	-
yes	0.48 *	0.28–0.82	0.80	0.55–1.17	0.72	0.46–1.13	0.80	0.50–1.15

Characteristics for item No. 1b and c: “less frequently” compared to “no changes”; for item No. 15: “no” compared to “yes”; and for item No. 13: “yes” compared to “no”. Bold and asterisk denote statistical significance.

## Data Availability

The data presented in this study are available on request from the corresponding author. The data are not publicly available due to patient confidentiality.

## Appendix A

**Table A1:** Description of 15 survey items.

Domains	No.	Item	Survey Periods (T)			
			1	2	3	4
Healthcare resource use	1	During the corona pandemic (current situation), the following has changed in my medical care. (If applicable, please indicate the changed frequency). Compared to before the pandemic, visits are less frequent/same/more frequent: (a) general practitioner, (b) neurologist, (c) physical therapy, (d) occupational therapy, (e) speech therapy, (f) rehabilitation sports.	x	x	x	
	**2**	The frequency of my medical care (general practitioner, neurologist) has not changed with the pandemic.				x
	**3**	The frequency of my therapeutic care (physiotherapy, occupational therapy, speech therapy, rehabilitation sports) has not changed with the pandemic.				x
	**4**	How often in the last three days have you been dissatisfied with your therapeutic care (physiotherapy, occupational therapy, speech therapy, rehabilitation sports)?				x
Perception and unmet needs	5	I miss important parts of my usual MS therapies.	x	x	x	x
	6	There are currently situations where I would need support, but I do not know where to go.	x	x	x	
	7	How often did it happen in the last few days that you were dissatisfied with the medical care you received for your MS?	x	x	x	x
	8	I would like to use medical advice more often during this time.	x	x	x	
	9	I would like to receive psychological support.	x	x	x	
	**10**	I have received or am receiving psychological support.				x
	11	I have thought about changing/discontinuing my MS therapy.	x	x	x	
	**12**	I stopped/changed my MS therapy because of the coronavirus.				x
	13	I would like to use telemedicine services and not meet the physician in a face-to-face situation.	x	x	x	
	**14**	I have been using telemedicine services so that I do not have to meet my physician in a face-to-face situation.				x
	15	I have already participated in internet-based sports training or similar.	x	x	x	

Items in bold were included in the survey over three (T1–T3) or four (T1–T4) survey periods. All others occurred exclusively in the last survey (T4) and reflect the previous periods of the pandemic. Survey periods (T), marked with an “x” if applicable: T1 = first survey from 6 April to 30 April 2020 (during the first COVID-19 wave: first lockdown); T2 = second survey from 8 July to 31 August 2020 (during the summer plateau, 2020: low incidence); T3 = third survey from 1 December 2020 to 31 January 2021 (during the second COVID-19 wave: partial lockdown and second lockdown; launch of the COVID-19 vaccination campaign in Germany); T4 = fourth survey from 8 October to 31 December 2021 (during the fourth COVID-19 wave: peak infections, expanded vaccination efforts, reintroduced public health measures).
